# Neural autoantibodies in psychiatric disorders are associated with antibodies against viral pathogens: a retrospective study of 619 patients

**DOI:** 10.1007/s00702-025-02943-x

**Published:** 2025-05-17

**Authors:** Niels Hansen, Vincent Buschatzky, Anne Katharina Bastin, Kristin Rentzsch, Bianca Teegen, Daniel Luedecke, Thomas Skripuletz, Hannah Benedictine Maier, Stefan Bleich, Jürgen Gallinat, Hermann Esselmann, Ildiko Rita Dunay, Inga Zerr, Dirk Fitzner, Jens Wilftang, Alexandra Neyazi, Björn Hendrik Schott, Berend Malchow, CAP (Cerebrospinal Fluid Analysis in Psychiatry) consortium

**Affiliations:** 1 Department of Psychiatry and Psychotherapy, University Medical Center Göttingen, Von-Siebold-Str. 5, 37075 Göttingen, Germany; 2 Clinical Immunological Laboratory Prof. Stöcker, Groß Grönau, Germany; 3 Department of Psychiatry and Psychotherapy, University Hospital Hamburg Eppendorf, Hamburg, Germany; 4 Department of Neurology, Hannover Medical School, Hannover, Germany; 5 Department of Psychiatry, Social Psychiatry and Psychotherapy, Hannover Medical School, Hannover, Germany; 6 Institute for Inflammation and Neurodegeneration, Otto-Von-Guericke-University Magdeburg, Magdeburg, Germany; 7 Center for Behavioral Brain Sciences, Otto-Von-Guericke-University Magdeburg, Magdeburg, Germany; 8 Department of Neurology, University Medical Center, Göttingen, Germany; 9 German Center for Neurodegenerative Diseases (DZNE), Von-Siebold-Str. 3a, 37075 Göttingen, Germany; 10 Neurosciences and Signaling Group, Institute of Biomedicine (iBiMED), Department of Medical Sciences, University of Aveiro, Aveiro, Portugal; 11 Department of Psychiatry and Psychotherapy, Otto-Von-Guericke-University Magdeburg, Magdeburg, Germany; 12 Leibniz Institute for Neurobiology, Magdeburg, Germany

**Keywords:** Autoimmunity, Autoantibodies, Psychiatric disorder, Viral antibody indices

## Abstract

A history of viral infection has been associated with a higher risk for psychiatric disorders. One potential underlying mechanism is that antiviral immunological responses could trigger cross-reactivity between viral and neural antigens, which would raise the co-occurrence of antiviral antibodies and anti-neural autoantibodies. We studied 619 patients’ psychiatric diagnoses from the Department of Psychiatry and Psychotherapy, University Medical Center Göttingen, Germany. Anti-neural autoantibodies and antiviral antibody specific indices were measured in serum and/or cerebrospinal fluid (CSF) from all patients. Among these 619 patients, 115 tested positive for serum and/or CSF neural autoantibodies (18.6%), with the most often identified autoantibodies being anti-GAD65 in serum (2.2%) and CSF (1.6%), and anti-NMDA in serum (0.6%) and CSF (1.3%). The three main diagnostic groups presenting neural autoantibodies were patients with organic psychiatric disorders including dementia (81 of 377; 21.7%), those with psychotic disorders (9 of 66; 13.6%), and patients with affective disorders (19 of 138; 13.9%). Logistic regression analysis revealed a significant association between the varicella zoster virus (VZV) antibody-specific index and autoantibody positivity in patients with all diagnoses (F00–F79) (p < 0.005). Furthermore, the rubella antibody-specific index proved to be significantly associated with neural autoantibody positivity (p < 0.001) across all patients (F00–F79), and in those with affective disorders (p < 0.01). Our results show that VZV and rubella antiviral antibodies are associated with a higher propensity to develop anti-neural autoantibodies, suggesting that the known association between viral infection and later developing psychiatric disorders may be partly attributable to the development of anti-neural autoimmunity.

**Supplementary Information:**

The online version contains supplementary material available at 10.1007/s00702-025-02943-x.

## Introduction

The potential association between infectious disease and psychiatric disorders has stimulated the interest of researchers for over a century, attracting renewed interest during the recent COVID-19 pandemic (Rybakowski 2022). Epidemiological evidence suggests that patients with a history of prior infections carry an increased risk for psychiatric disorders (Blomström et al. 2014; Sutterland et al. 2015), most prominently psychotic conditions like schizophrenia (Jiang et al. 2020; Dalman et al. 2020; Benros et al. 2011, 2012), but also affective disorders like major depressive disorder (MDD) and bipolar disorder (BD) (Sutterland et al. 2015; Benros et al. 2013), as well as cognitive disorders including dementia (Chiu et al. 2014; Tzeng et al. 2018; Scherrer et al. 2022). Numerous mechanisms have been proposed to explain these associations, including detrimental effects of pro-inflammatory cytokines, dysregulated tryptophan/kynurenine metabolism, and the production of autoantibodies against neural structures dementia (Müller 2014; Prüss 2021; Schwenkenbecher et al. 2021).

The increased prevalence of neural autoantibodies has been described in conjunction with various psychiatric anomalies including psychotic disorders (Endres et al. 2022; Daguano Gastaldi et al. 2023; Jeppesen et al. 2023), affective disorders (Endres et al. 2022; Daguano Gastaldi et al. 2023; Grenzer et al. 2022) or cognitive disorders (Endres et al. 2021; Hansen et al. 2022; Juhl et al. 2022). With the few exceptions of rare psychiatric conditions directly attributable to autoantibody-related encephalitis (Graus et al. 2016), (especially NMDA receptor encephalitis (Peery et al. 2012), the pathophysiological relevance of neural autoantibodies still needs to be clarified. The endeavor to establish a mechanistic link between neural autoantibodies and psychiatric disease is further challenged by the fact that neural autoantibodies have been detected in patients with affective and schizophrenia-spectrum disorders, those with neurodegenerative and autoimmune diseases, but also in healthy control participants (Daguano Gastaldi et al. 2023].

To assess a potential relationship between antiviral antibody indices and neural autoantibodies, it would therefore make sense to focus on cerebrospinal fluid (CSF) biomarkers, which are probably more closely related to cellular central nervous system (CNS) pathology than are antibodies in peripheral blood. In the present study, we examined CSF and serum samples from inpatients and outpatients of a university-based psychiatric hospital for the presence of neural autoantibodies. We then tested whether elevated antibody-specific indices to viral pathogens would differentiate psychiatric patients from those without neural autoantibodies.

## Methods

### Patient recruitment

A total of 691 patients from our Department of Psychiatry and Psychotherapy at the University Medical Center Göttingen were recruited cross-sectionally in 2021 and 2022. The data from 72 patients were excluded from analysis because they were follow-up patients. Our final study sample thus consisted of 619 patients. The International Classification of Diseases 10 th edition (ICD-10) was used to classify patients’ psychiatric diagnoses (WHO 2020). Their records were examined retrospectively and their primary and secondary psychiatric diagnoses recorded. We further documented other comorbid diagnoses such as a recent or current Corona Virus Disease 2019 (COVID-19) infection, type 2 diabetes mellitus, current or past neoplasms, and rheumatologic disease. This study was carried out in accordance with the current version of the Declaration of Helsinki (World Medical Association 2013) and approved by the Ethics Committee of the University Medical Center Göttingen (AZ1/6/20).

### Cerebrospinal fluid diagnostics

All patients underwent lumbar puncture as part of their routine clinical diagnostic work-up and consented to the research use of their laboratory results. The CSF samples were examined at the Neurochemical Laboratory of the Department of Neurology of the University Medical Center Göttingen.

#### Routine CSF laboratory exams

Using routine clinical laboratory diagnostics, the parameters we determined in the CSF from all patients were: cell count per µl, percentage of lymphocytes, monocytes and granulocytes (relative to total CSF leukocytes), as well as lactate, total protein, albumin, immunoglobulin G (IgG), immunoglobulin M (IgM) and immunoglobulin A (IgA), and oligoclonal bands. Albumin, IgG, IgA, and IgM parameters were also measured in the serum on their lumbar-puncture day, as were quotients (Albumin, IgG, IgA, IgM) from cerebrospinal fluid and serum.

#### Antiviral antibody specific indices

We determined antibody values of IgG and IgM in serum and CSF against many viral antigens [varicella zoster virus (VZV), herpes simplex virus 1/2 (HSV1/2), Epstein Barr virus (EBV), cytomegaly virus (CMV), measles and rubella] as well as viral antibody specific indices (VZV, HSV, EBV, CMV, measles and rubella) were calculated to determine potential intrathecal synthesis***.***

#### Neurodegeneration markers

We measured CSF biomarkers of neurodegeneration, particularly dementia attributable to Alzheimer´s disease [total tau (t-tau), phosphorylated tau protein 181 (p-tau181), amyloid-beta 40 (Aβ40), amyloid-beta 42 (Aβ42), and the amyloid beta 42/amyloid beta 40 (Aβ42/40) ratio]. Concentrations of t-tau and p-tau181 levels we determined via ELISAs from Fujirebio (Tokyo, Japan) (Innotest, hTau Ag and Innotest: p-tau 181). To quantify amyloid beta peptides, we ran immunoassays from Fujirebio [INNOTEST^®^ β-amyloid (1–42) ELISA) and IBL (Amyloid beta (1–40)]. CSF markers of neural cell damage, included S100 and neuron-specific enolase (NSE). NSE and S100 concentrations in serum were likewise examined, as were viral antibody values.

### Neural autoantibody determination

Neural autoantibodies of all patients were analyzed at the Neurochemistry Laboratory of the Department of Neurology, University Medical Center Göttingen and at the Clinical Immunological Laboratory (Groß Grönau, Germany) as part of the standard clinical CSF examination. Both BIOCHIPS mosaics with brain tissue and recombinant cell preparations were used to identify IgG autoantibodies. Specifically, we used standard immunofluorescence assays with additional cell-based assays for Hu, Ri, Yo, Tr/DNER, Ma/Ta, glutamic acid decarboxylase 65 (GAD65), amphiphysin, aquaporin 4, myelin oligodendrocytic protein (MOG), N-methyl-D-aspartate receptor (NMDAR), α-amino-3-hydroxy-5-methyl-4-isoxazolepropionic acid receptor 1/2 (AMPAR1/2), gamma aminobutyric acid receptor B (GABABR), Leucin rich glioma inactivated protein 1 (LGI1), contactin-associated protein-2 (CASPR2), IgLON5, titin, Zic4, dipeptidyl peptidase-like protein 6 (DPPX) with a cut-off positivity of 1:10. We also ran standard immunofluorescence assays for ANNA3 with an autoantibody-positivity threshold of 1:10, and antimyelin antibodies with an antibody-positivity threshold of 1:100. We ran separate cell-based assays for glycine receptors, recoverin, adaptor-related protein complex 3 subunit beta 2 (AP3B2), neurofascin 186, Purkinje cell protein carbonic anhydrase-related protein VIII (CARPVIII), KCNA2, GABAAR, antibodies against Rho GTPase-activating protein 26 (ARHGAP26), and flotillin 1/2 antibodies with 1:10 autoantibody positivity. We also assessed Homer 3, GFAP, neurochondrin, and neurexin3alpha via cell-based and immunofluorescence assays at 1: 100 cut-off positivity.

### Statistical analysis

We used Sigma Plot (Version 11.0) to construct graphs and Sigma Stat (Version 11.0) for statistical analysis. Comparisons between autoantibody-negative and -positive patients were done by Fisher’s exact test for differences between sex, clinical comorbidities, frequencies of psychiatric diagnoses, the presence of an intrathecal IgG synthesis (score of an intrathecal IgG synthesis: intrathecal IgG synthesis: score 1, intrathecal IgG synthesis absent: score 0) and any blood–brain-barrier disturbances (score of a blood–brain-barrier disturbance: blood–brain barrier disturbance present: score 1, blood–brain-barrier disturbance absent: score 0). All other comparisons between diagnostic groups and between antibody-positive and -negative patients were made via Mann–Whitney *U* tests when data were not normally distributed and with two sample t-tests when normal distribution could be assumed. Normal data-distribution was subjected to the Shapiro Wilk test. We calculated logistic regressions for all CSF parameters including antibody indices related to antibody status (positive = 1, negative = 0). A significance level of p < 0.05 was considered as significant. Bonferroni correction for multiple comparisons was applied for demographic data (two parameters: sex and age), comorbidities (four parameters: diabetes mellitus, COVID19, rheumatologic disease and tumor; adjusted p < 0.012), diagnostic categories (eight parameter: F00–09, F10–19, F20–29, F30–39, F40–49, F50–59, F60–69, F70–F79; adjusted p < 0.006), CSF cell parameters [four parameters: cell count, percentual number of lymphocytes, monocytes, granulocytes; adjusted p < 0.013], CSF protein parameter [eight parameters: CSF/serum ratio of IgG, IgA, IgM; albumin, albumin CSF/serum ratio, total protein; the presence of intrathecal IgG antibody synthesis and any blood–brain-barrier disturbance; adjusted p < 0.006] and CSF antibody indexes (five parameters: VZV, HSV, EBV, measles and rubella; adjusted p < 0.01).

## Results

### Demographics of the study cohort

Our cohort included 619 psychiatric patients recruited from inpatient and outpatient units of the Department of Psychiatry at the University Medical Center Göttingen. Our cohort included patients with a wide range of psychiatric diagnoses including the diagnostic groups F00–F09 (organic, including symptomatic, mental disorders), F10–F19 (mental and behavioral disorders caused by psychoactive substance use), F20–F29 (schizophrenia, schizotypical and delusional disorders), F30–39 mood [affective] disorders, F40–49 (neurotic, stress-related and somatoform disorders), F50–F59 (behavioral syndromes associated with physiological disturbances and physical factors), F60–F69 (unspecified disorder of adult personality and behavior), and F70–F79 (intellectual disabilities). The main diagnoses, sex, age and frequencies of main diagnoses of patients are shown in supplement Table 1 and Table 1 and did not differ between autoantibody-positive and autoantibody-negative patients. A minority (n = 12 of 115 antibody-positive patients, 10.44%) of psychiatric patients underwent immunotherapy with methylprednisolone, intravenous immunoglobulins, mycophenolate mofetil, plasmapheresis and/or azathioprine. The clinical outcome was predominantly an improvement or stabilization in 9 of 12 (75.19%) treated patients; worsening symptoms were observed in 2 of 15 (16.67%) treated patients. Tumors were reported in 16 of 115 (13.9%) antibody-positive patients (see also supplement Table 1).

**Table 1:** Diagnoses of patients

	F00–F09	F10–F19	F20–F29	F30–F39	F40–F49	F50–F59	F60–F69	F70–F79
Autoantibody positive	81/115 (70.43%)	3/115 (2.61%)	8/115 (6.96%)	19/115 (16.52%)	4/115 (3.48%)	0/115 (0%)	0/115 (0%)	1/115 (0.86%)
Autoantibody negative	296/504 (58.73%)	12/504 (2.38%)	57/504 (11.31%)	119/504 (23.61%)	16/504 (3.17%)	2/504 (0.39%)	0/504 (0%)	1/504 (0.19%)
Statistics	p < 0.05#	p = 0.748	p = 0.236	p = 0.107	p = 0.775	p = 1	p = 1	p = 0.185

In each diagnosis category the number of patients from all autoantibody positive (n = 115) or negative (n = 504) are depicted as numbers and in brackets as percentages. For statistical analysis Fisher´s exact tests were used

#Not significant due to Bonferroni correction

### Detection of neural autoantibodies in the main cohort

Within our cohort, we identified 115 patients positive for neural autoantibodies in serum and/or CSF (18.6%). Three patients were examined in a follow-up visit so that their samples were counted twice in the total sample. Autoantibody-positive patients were significantly older (mean age: 67.6 ± 17.7 years) than autoantibody-negative patients (mean age: 62.9 ± 17.6 years; Mann Whitney *U* test: U = 23,957, p < 0.005, Table 1 supplement). The data on both groups’ specific and unspecific neural antibodies are in Table 2. The most frequent autoantibodies against intracellular antigens were anti-GAD65 in serum (2.2%) and CSF (1.6%), and the most frequent autoantibodies against cell-surface antigens were anti-NMDAR antibodies in serum (0.6%) and CSF (1.3%) (Table 2). The detailed frequencies of specific neural autoantibodies in the three diagnostic groups are shown in Table 2. Furthermore, the not-routinely assessed neural autoantibodies listed below were detected in diagnostically homogeneous groups without our knowing the tested frequency of antibodies in groups (see Table 2 supplement). Autoantibody-positive and -negative patients did not differ in baseline clinical characteristics like sex, diagnoses, recent COVID19 infection, diabetes mellitus type 1 or 2, history of or current neoplasm or inflammatory rheumatologic disease (Table 1 supplement). We observed no differences in the frequency of diagnostic groups between autoantibody-positive and -negative patients.

**Table 2:** Presence of standard specific neural autoantibodies in patient cohorts

Neural autoantibody	F00–F79	F00–F09	F20–F29	F30–F39
Intracellular antigens				
Anti-GAD65 CSF	8/493 (1.6%)	3/302 (1%)	1/54 (1.8%)	3/110 (2.7%)
Anti-GAD65 Serum	13/544 (2.3%)	6/326 (1.8%)	1/59 (1.7%)	5/127 (3.9%)
Anti-SOX1 CSF	3/480 (0.63%)	1/294 (0.3%)	0/54 (0%)	2/106 (1.9%)
Anti-SOX1 Serum	7/542 (1.3%)	5/324 (1.5%)	0/59 (0%)	2/127 (1.6%)
Anti-Ma2 CSF	1/493 (0.2%)	1/302 (0.3%)	0/54 (0%)	0/110 (0%)
Anti-Ma2 Serum	3/544 (0.55%)	2/326 (0.6%)	0/59 (0%)	0/127 (0%)
Anti-Ma1 CSF	0/6 (0%)	0/2 (0%)	0/3 (0%)	0/110 (0%)
Anti-Ma1 Serum	0/7 (0%)	0/2 (0%)	0/3 (0%)	0/127 (0%)
Anti-APH CSF	2/491 (0.4%)	2/300 (0.7%)	0/55 (0%)	0/106 (0%)
Anti-APH Serum	3/544 (0.6%)	3/326 (0.9%)	0/59 (0%)	0/127 (0%)
Anti-CV2 CSF	0/480 (0%)	0/294 (0%)	0/54 (0%)	0/109 (0%)
Anti-CV2 Serum	3/542 (0.6%)	2/324 (0.9%)	0/59 (0%)	0/127 (0%)
Anti-Ri CSF	0/493 (0%)	0/303 (0%)	0/54 (0%)	0/109 (05)
Anti-Ri Serum	0/544 (0%)	0/326 (0%)	0/59 (0%)	0/127 (0%)
Anti-Yo CSF	4/491 (0.8%)	3/301 (1%)	1/54 (1.8%)	0/109 (0%)
Anti-Yo Serum	8/544 (1.5%)	5/326 (1.6%)	1/59 (1.7%)	1/127 (0.8%)
Anti-HuD CSF	1/495 (0.2%)	1/303 (0.3%)	0/54 (0%)	0/110 (0%)
Anti-HuD Serum	0/545 (0%)	0/326 (0%)	0/59 (0%)	0/128(0%)
Anti-Zic4 CSF	4/493 (0.8%)	3/303 (0.1%)	0/54 (0%)	0/109 (0%)
Anti-Zic4 Serum	4/547 (0.7%)	3/326 (0.9%)	0/59 (0%)	0/127 (0%)
Anti-Tr CSF	0/497 (0%)	0/302 (0%)	0/54 (0%)	0/110 (0%)
Anti-Tr Serum	0/542 (0%)	0/326 (0%)	0/59 (0%)	0/127 (0%)
Cell surface autoantibody				
Anti-NMDAR CSF	3/499 (0.6%)	3/306 (1%)	0/56 (0%)	0/110 (0%)
Anti-NMDAR Serum	7/550 (1.3%)	6/329 (1.8%)	0/61 (0%)	1/128 (0.8%)
Anti-LGI1 CSF	0/500 (0%)	0/306 (0%)	0/56 (0%)	0/100 (0%)
Anti-LGI1 Serum	4/550 (0.7%)	2/329 (0.6%)	1/61 (1.6%)	0/128 (0%)
Anti-AMPAR1 CSF	0/491 (0%)	0/302 (0%)	0/55 (0%)	0/108 (0%)
Anti-AMPAR1 Serum	0/554 (0%)	0/329 (0%)	0/61 (0%)	0/128 (0%)
Anti-AMPAR2 CSF	0/491 (0%)	0/302 (0%)	0/55 (0%)	0/108 (0%)
Anti-AMPAR2 Serum	0/550 (0%)	0/329 (0%)	0/61 (0%)	0/128 (0%)
Anti-DPPX CSF	0/492 (0%)	0/300 (0%)	0/55 (0%)	0/108 (0%)
Anti-DPPX Serum	0/550 (0%)	0/329 (0%)	0/61 (0%)	0/128 (0%)
Anti-CASPR2 CSF	0/498 (0%)	0/303 (0%)	0/56 (0%)	0/112 (0%)
Anti-CASPR2 Serum	3/550 (0.5%)	3/329 (0.9%)	0/61 (0%)	0/128 (0%)
Anti-Aquaporin 4 CSF	0/34 (0%)	0/15 (0%)	0/8 (0%)	0/9 (0%)
Anti-Aquaporin 4 Serium	0/36 (0%)	0/15 (0%)	0/7 (0%)	0/12 (0%)
Anti-GABAB1/2 CSF	0/499 (0%)	0/306 (0%)	0/56 (0%)	0/112 (0%)
Anti-GABAB 1/2 Serum	2/550 (0.4%)	2/329 (0.6%)	0/64 (0%)	0/128 (0%)

*AMPAR1/2* alpha-amino-3-hydroxyl-5-methyl-4-isoxazole propionic acid receptor 1/2, *APH* amphiphysin, *CASPR2* Contactin-associated protein-like 2, *CSF* cerebrospinal fluid, *CV2* cronveinten 2, *DPPX* dipeptidyl-peptidase-like protein 6, *GABABR* Gamma-aminobutyric Acid Type B Receptor, *GAD65* glutamic acid, decarboxylase 65, *LGI1* leucin rich glioma inactivated 1 protein, *NMDAR* N-methyl-D-aspartate-receptor

### Detection of neural autoantibodies in our subcohorts

We formed three main diagnostic groups (1: F00–F09, 2: F20–F29 and 3: F30–F39) to assess potential group-specific differences between autoantibody-positive and -negative patients: among the F00–F09 group, 81 of 377 patients (21.7%) presented neural autoantibodies, among the F20–F29 group 9 of 66 (13.6%) patients did as well; autoantibodies were also detected in 19 of 138 patients (13.9%) among the F30–F39 group of disorders (in Table 1 all diagnostic groups are shown). No differences between diagnostic categories emerged in all patients (Table 1). The detailed frequencies of specific neural autoantibodies in the three diagnostic groups are shown in Table 2 and the Table 2 supplement, respectively. The diagnostic subgroups of antibody-positive and -negative patients did not differ in their baseline clinical parameters such as age, sex, frequency of secondary diagnoses, and comorbid diagnoses, e.g., diabetes mellitus type 2, recent COVID19 infection, tumor, or rheumatologic disease (Table 1 supplement). We did not subdivide the subgroups F00–F09, F20–F29 and F30–39 into additional individual diagnostic categories, as that would have made our sample sizes too small.

### CSF clinical parameter analysis of cells, proteins, and neuronal cell damage markers

We observed no statistically significant differences among the following parameters: total protein count, albumin, IgG CSF/serum ratio, IgA CSF/serum ratio, IgM CSF/serum ratio or the presence of oligoclonal bands among patient groups comparing autoantibody-negative to -positive patients when considering all diagnostic and subgroups together (F00–F79) (Table 3 supplement). However, note that we detected intrathecal IgG synthesis more frequently in patients with F00–F09 as well as neural autoantibodies (14.08%, 10 of 71 patients) than in patients with F00–09 who presented no autoantibodies (1.79%, 5 of 280 patients, Fisher´s exact test, p < 0.001, Table 3 supplement). In addition, there were no differences in cell count and relative frequencies of lymphocytes, granulocytes, or monocytes between autoantibody-positive and -negative patients in our main cohort (F00–F79) and in subcohorts with homogeneous diagnostic groups (1: F00–F09, 2: F20–F29, 3: F30–F39) (Table 3 supplement). CSF dementia biomarkers (t-tau protein, p-tau181, NSE, S100, Aβ42, Aβ40, Aβ42/Aβ40 ratio) failed to differ either between autoantibody-positive and -negative patients in any group (Table 3 supplement).

### Association between neural autoantibodies and antiviral antibody indices

**Fig. 1:**
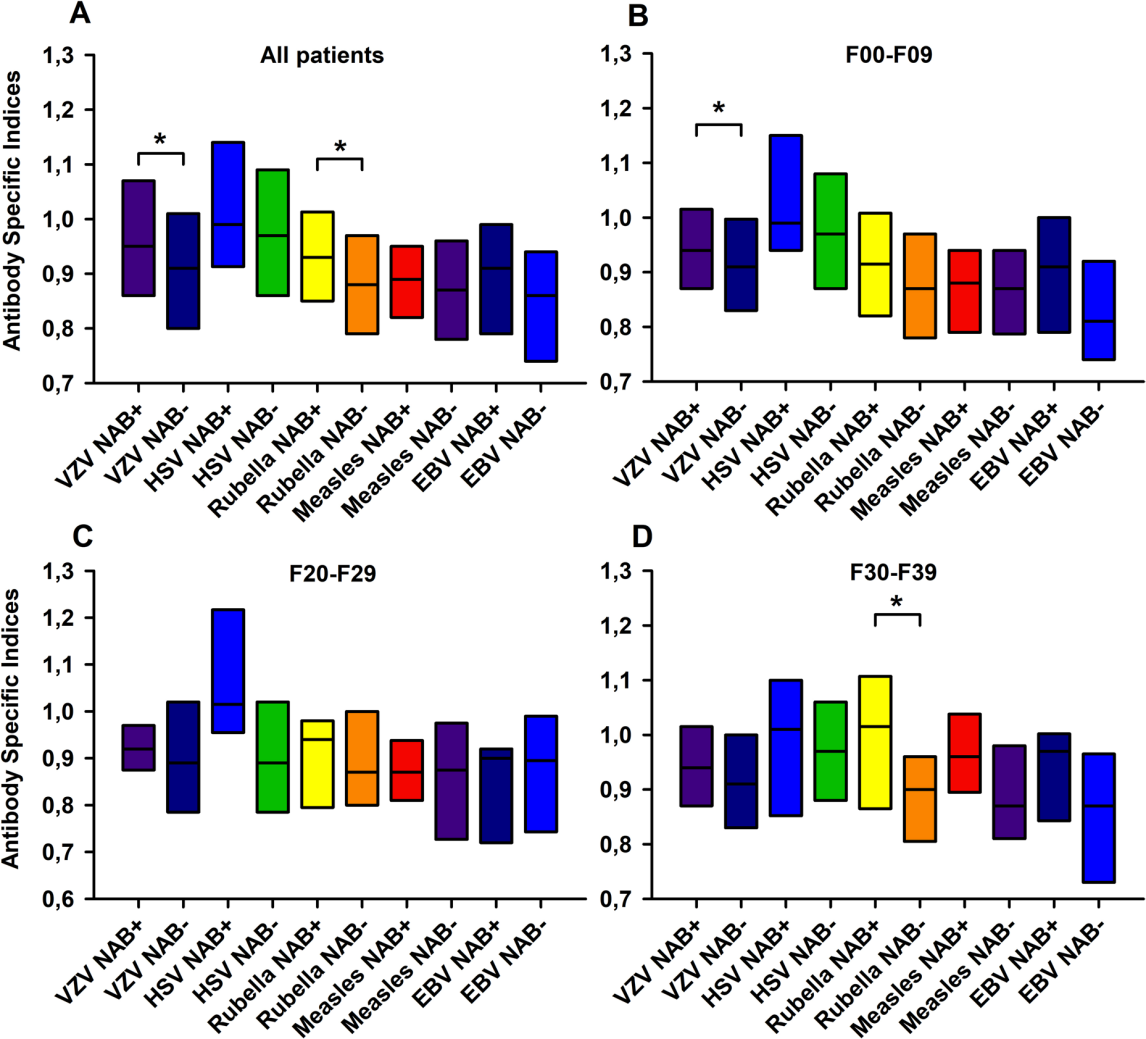
Higher antibody indices against VZV and rubella in psychiatric patients. We detected increased antibody specific indices for VZV (**A**) (p < 0.005) and the rubella antibody specific index (p < 0.001) in neural autoantibody-positive versus negative groups in all patients in diagnostic groups F00–F79. In addition, the VZV antibody specific index was higher (p < 0.001) in autoantibody-positive psychiatric patients diagnosed F00–F09 than in autoantibody-negative patients (**B**). Furthermore, in (**D**) we detected a higher rubella antibody specific index (p < 0.01) in autoantibody-positive psychiatric patients diagnosed F30–F39 than in autoantibody-negative patients. Patients diagnosed F20–F29 with neural autoantibodies revealed no increased viral pathogen antibody specific index (**C**). *HSV* herpes simplex virus, *VZV* varicella zoster virus, *EBV* Ebstein Barr virus, *NAB+* neural autoantibody positive, *NAB–* neural autoantibody negative

**Fig. 2:**
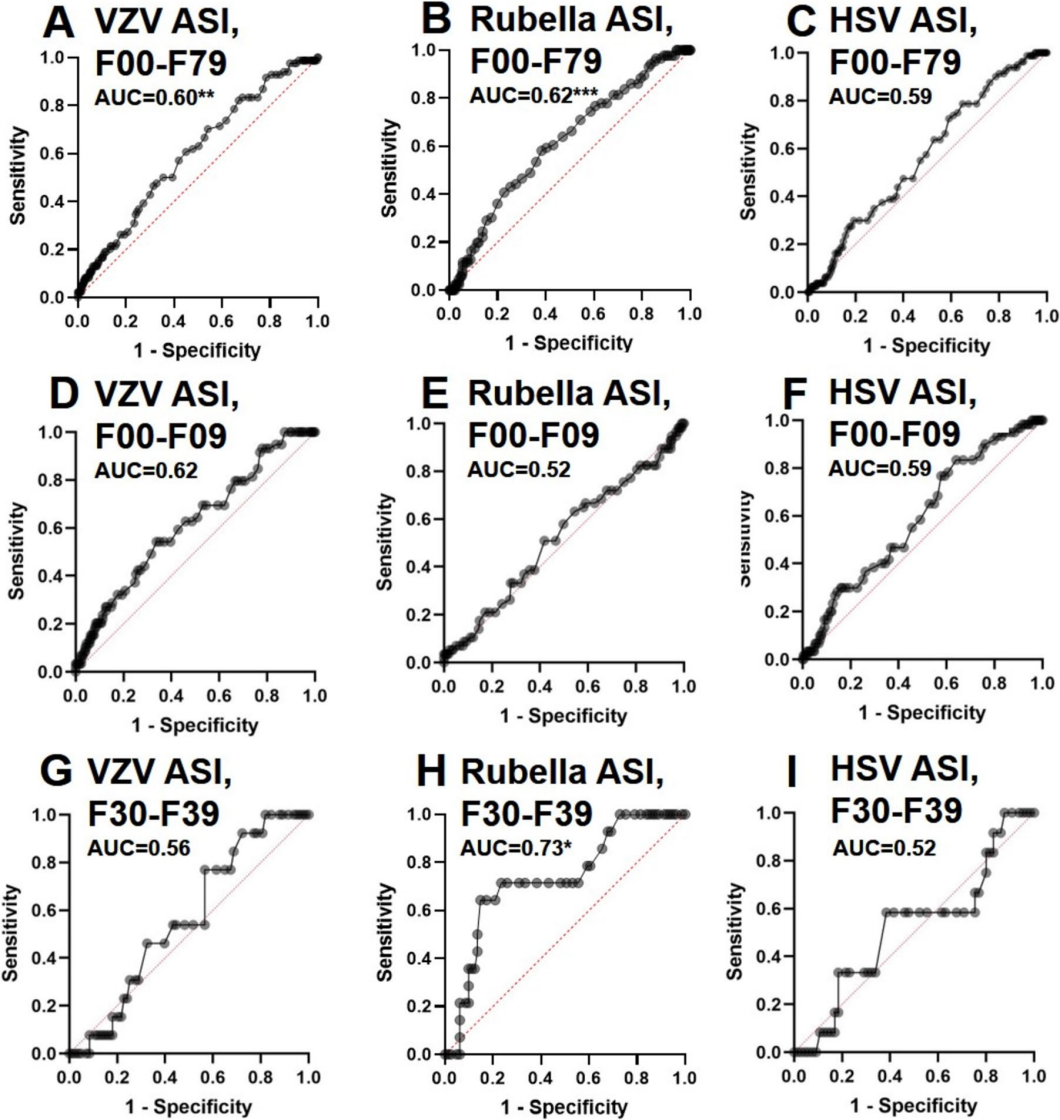
Logistic regression analysis of patients with antibody indices against viral antigens. The VZV antibody index showed a significant regression predicting neural autoantibody-positivity with an AUC of 0.60 (p < 0.005) for all diagnostic groups (F00–F79) (**A**). In addition, the rubella antibody specific index also showed relevant regression predicting autoantibody positivity in patients from all diagnostic groups with an AUC of 0.62 (F00–F79) (p < 0.001) (**B**). Interestingly, the rubella antibody specific index also showed an even higher regression with an AUC of 0.73 to distinguish autoantibody-positive from -negative patients (p < 0.01) (**H**). *ASI* antibody specific index, *AUC* area under the curve, *HSV* herpes simplex virus, *VZV* varizella zoster virus

Antibody values against common viruses (VZV, HSV, EBV, CMV, rubella, and measles) in CSF and serum samples depicted as quotients did not differ between our autoantibody-positive and -negative diagnostic subgroups (Table 3 supplement). However, antibody-specific indices against VZV and rubella proved to be significantly higher in patients with autoantibodies than in those with none (VZV antibody specific index: Mann Whitney U test, p < 0.005, U = 13,924; rubella antibody specific index: Mann Whitney U test, p < 0.005, U = 12,262, Fig. 1A, the corresponding numbers of patients tested for antibody-specific indices of IgG for Fig. 1 are shown in the Table 4 in supplement). When considering only patients with organic psychiatric disorders (F00–F09, Fig. 1B), we noted significantly higher VZV antibody-specific indices in the autoantibody-positive than autoantibody-negative patients (Mann Whitney U test, p < 0.001, U = 3866) (Fig. 1B). We also observed higher anti-rubella-antibody-specific indices for rubella in autoantibody-positive than in autoantibody-negative patients in the affective disorders group (F30-F39, Mann Whitney U test: p < 0.01, U = 302, Fig. 1D). Logistic regression analysis revealed that the VZV antibody-specific index in patients with all diagnoses (F00–F79) separated autoantibody-positive from autoantibody-negative patients significantly (AUC = 0.60, confidence interval 0.53–0.66, p < 0.005, best fit values: β0 = – 1.77, β1 = 0.27, X at 50% 2.66; Fig. 2A). The specific anti-rubella antibody-specific index also distinguished significantly between autoantibody-positive and autoantibody-negative patients (logistic regression: AUC = 0.62, confidence interval 0.55–0.68, p < 0.001, best fit values: β0 = – 2.09, β1 = 0.73, X at 50% = 2.83; Fig. 2B) in all patients (F00-F79) and in those with only depressive disorders (F30–F39, logistic regression: AUC = 0.73, confidence interval 0.59–0.87, p < 0.01, best fit values: β0 = – 4.24, β1 = 2.60, X at 50% = 1.62; Fig. 2H). Multiple regression analysis showed only a slight improvement in AUC when accounting for both specific VZV- and rubella antibody-specific indices [AUC = 0.65, p < 0.0001, best fit values: β0 = – 2.2, β1 = 0.24 (VZV antibody-specific index), β2 = 0.54 (rubella antibody-specific index), data not shown]. The other logistic regression analyses revealed no significant associations (see Fig. 2C–G, I).

## Discussion

The present study provides evidence for an association between antiviral-specific antibody indices and neural autoantibodies in the CSF and serum of patients with psychiatric disorders. Specifically, our findings suggest that the anti-VZV and anti-rubella antibody-specific indices are associated with the presence of neural autoantibodies in patients with psychiatric conditions, with preferential associations between anti-VZV and neural autoantibodies in organic psychiatric disorders, and between anti-rubella and neural autoantibodies in patients with affective disorders.

### Varicella zoster virus and organic psychiatric disorders

Our results suggest that a VZV infection history is associated with a stronger likelihood of expressing neural autoantibodies in patients with organic psychiatric disorders, although this is hypothetical. To establish a plausible link between the occurrence of a VZV infection and, for example, a subsequent autoimmune psychosis, it is important to demonstrate a temporal association if the VZV infection serves as the trigger for a subsequent autoimmune reaction. Our study has no such data available, as we do not know when a patient contracted a viral VZV infection, making it therefore impossible to postulate such a temporal association from our data. While dermatologic and peripheral nerve sequelae are the most frequent manifestations of VZV infection and re-infection, evidence also supports the notion that VZV is a key infectious agent in encephalitis (Eckerström et al. 2020; Liem et al. 2023), a property shared by other viruses from the extended herpes virus family, such as herpes simplex virus (HSV) 1 and 2, cytomegaly virus (CMV), and Epstein-Barr virus (EBV) (Liem et al. 2023; Watson et al. 2013; Dyachenko et al. 2018; Yamamoto and Nakamura 2000). Both VZV and HSV infections are known risk factors for dementia (Shim et al. 2022), although more research is needed (Warren-Gash et al. 2019). Along the same line, HSV (and to a lesser extent VZV also) have been associated with cognitive dysfunction (Watson et al. 2013; Grahn et al. 2013; Fruchter et al. 2015). The association between the anti-VZV antibody-specific index and presence of neural autoantibodies in patients with organic psychiatric disorders especially as in this study suggests that VZV and potentially other herpes virus subtypes may trigger their adverse effects on cognitive functioning and the dementia risk by promoting autoimmunity in the CNS. This may be a poly-specific reaction within the context of chronic inflammation, such as that observed in neurological diseases like multiple sclerosis and neuropsychiatric diseases such as autoimmune encephalitis. In line with this interpretation, elevated levels of astrocyte activation and neurodegeneration markers (glial fibrillary acidic protein, GFAP; neurofilament light chain, NfL) have been identified in patients with VZV encephalitis (Grahn et al. 2013). Note, however, that we failed to observe any significant association between the neural autoantibody status and anti-HSV antibody-specific index. In patients with psychotic disorders (F00-F29), however, we did detect nominally higher anti-HSV antibody-specific indices in neural autoantibody-positive than in -negative patients (Figs. 1, 2) – it is, in our view, highly probable that this difference will prove to be significant in a larger cohort, a finding that would concur with the evidence of an association between HSV infection and cognitive impairment in schizophrenia (Dickerson et al. 2012, 2016).

### A potential role for the rubella virus in affective disorders

While the association we detected between VZV’s antiviral antibody-specific index and the presence of neural antibodies is clearly in line with previous research, our finding that the anti-rubella antibody index differentiated psychiatric conditions, particularly affective disorders with and without autoantibodies, was surprising. With the well-known exception of severe neurodevelopmental disorders resulting from maternal infection during pregnancy (Mawson and Croft 2019; Al-Haddad et al. 2019; Gordon-Lipkin et al. 2021), rubella-associated CNS diseases are rare, and few if any associations between the rubella antibody status and psychiatric conditions have been reported so far. As herpes viruses do, however, the rubella virus shares common peptides with the distal-less homeobox (DLX) proteins critical for CNS development (Lucchese and Stahl 2018). As vaccination rates against the rubella virus are high in industrialized countries (Lambert et al. 2015), we cannot rule out that the association between neural autoantibodies and the anti-rubella antibody index may be attributable to vaccination. This interpretation would be in line with the observation of rare adult rubella encephalitis following vaccination (Gualberto et al. 2013) and with the temporal association between vaccinations and the onset of several psychiatric disorders like obsessive–compulsive disorder or anorexia nervosa (Leslie et al. 2017). However, the preferential association we have revealed between the anti-rubella antibody index and neural autoantibodies in affective disorders is, to our knowledge, novel and warrants further investigation.

### Infections and CNS autoimmunity

The syndrome-specific associations between the antibody indices of viral antigens and autoantibody titers support the importance of anti-viral immunity in developing neural autoimmunity in psychiatric illness. In line with this notion, a recent study of 100 chronically psychotic patients reported an elevated EBV antibody index in those individuals (Runge et al. 2022). As EBV was not assessed in enough patients in our cohort, we cannot draw conclusions on a potential association between the EBV antibody index and neural autoantibodies. There is evidence that a viral brain infection can lead to conditions triggering autoimmunity, i.e., herpes simplex virus encephalitis (Armangue et al. 2014, 2018), or SARS-Cov2-related autoimmunity in the brain (Gupta and Weaver 2021; Franke et al. 2023). Recent data suggest that specific genetic factors such as human leukocyte antigen (HLA) haplotypes may play a role when a virus triggers an autoimmune disease in the brain (Joubert and Dalmau 2019). The potential role of infection-triggered autoimmunity in the development of psychiatric disorders is further highlighted by the observation that, in a mouse model, toxoplasma-induced neuroinflammation elicited weaker expression of multiple synaptic proteins that were implicated in the pathogenesis of psychiatric disorders via genome-wide association studies (Lang et al. 2018). Several mechanisms are known to induce an autoimmune response triggered by infection, such as cross-reactive T cells in molecular mimicry reactions and bystander T-cell activation with epitopes spreading, leading to a T-cell-dominated autoimmune response (Getts et al. 2013). Unlike neuronal autoantibodies, T-cell-mediated autoimmunity is challenging to study in routine clinical practice, but abnormalities in T-cell populations in psychiatric disorders should be investigated in future studies (Garza et al. 2023).

### Intracellular autoantibodies and cancer immunity

It is also important to mention that a large proportion of the detected antibodies were those against intracellular antigens and are therefore often paraneoplastic. The tumors reported in history are known and present in 13.9%, but we cannot rule out that new or current tumors are present. A whole-body fluorodeoxyglucose positron emission tomography (FDG PET) of antibody-positive patients would have been necessary to rule this out. In addition, tumors can also develop more frequently with membrane surface autoantibodies, as a study recently demonstrated (Kerstens et al. 2024); a complex diagnosis involving an FDG PET of the entire body would also have had to be carried out in such patients to rule out tumors. But again, as this study is retrospective, that is not possible. This would, however, be essential in a future study to answer the key question of whether it is a case of cancer immunity or an autoimmune reaction. This question cannot be unequivocally answered in this study. Intracellular antibodies that are rarely associated with tumors are the GAD65 autoantibodies—the most frequently reported intracellular antibody group. Nevertheless, note also that a high antibody titer of GAD65 autoantibodies is known to play a major role in their pathogenicity (Budhram et al. 2021). Nevertheless, we are also aware that a GAD65 titer does not define the disease phenotype per se (Moura et al. 2024). In assessing our GAD autoantibodies, we conducted purely semi-quantitative evaluations: slightly to strongly positive were carried out without calculating an exact titer. These titer calculations cannot therefore serve to assess the pathogenicity in our study.

### Limitations

One inherent study limitation is the heterogeneity of neural autoantibodies and limited informative potential of a heterogeneous group of autoantibodies blocking different target antigens at the cell surface and in the intracellular space. On the other hand, it is also possible that a non-specific viral reaction acts as a trigger for subsequent autoantibody production regardless of the antibody subtype. In addition, our diagnostic subgroups were small and showed considerable variability, meaning statistical power was limited, and we could only assess associations in larger diagnostic categories but not subgroups. Nevertheless, the possibility of pooled testing in different diagnostic groups offers the opportunity to investigate the fundamental relationship between a patient’s neural autoantibody positivity and their anti-viral specific antibody index with more certainty thanks to significantly higher number of patients. This also tends to apply to cognitive disorders, but less so to affective and psychotic disorders. Furthermore, our sample included patients of different diagnostic categories, but no healthy control cohort, as lumbar punctures in healthy individuals would have been too invasive for a purely research purposes. We therefore have an antibody-positive group and a disease control group presenting no evidence of antibodies, and the latter group should be regarded as the control group in our retrospective study design. As we have an antibody-negative disease control group available, conclusions on a causal link are possible in relation to mental illness without autoantibodies, but not in relation to mental health without or with autoantibodies, so these limitations should be considered when interpreting our results. As our study is retrospective, we are unable to determine exactly when the mental disorders first occurred and during which period the anti-viral and anti-neural antibodies were determined. A clear assessment over time is therefore not possible. Nevertheless, we examined a large panel of neural autoantibodies in a relatively large sample of psychiatric patients, uncovering thereby an association between antiviral immunity and autoimmunity in patients with psychiatric conditions. Nevertheless, it is important to point out that only associations and no causal demonstration between neural autoantibodies and anti-viral antibodies were made. It would be interesting for a future research project to show the cross-reactivity between anti-neural autoantibodies and anti-viral antibodies in inhibition experiments.

### Conclusions

The results of our study suggest that the antibody-specific indices of viral pathogens are associated with the presence of neuronal autoantibodies in psychiatric disorders. Future research should follow up our findings by investigating the role of antiviral antibody-specific indices, such as VZV in autoantibody-related cognitive impairment, and the specific rubella antibody-specific index in affective disorders in larger, homogeneous subcohorts to better elucidate their role in disease pathogenesis and autoantibody production. The timing of the development of autoimmunity also needs further clarification, as a recently published study (Liu et al. 2023) suggests that autoimmunity may develop early in the acute viral encephalitis stage.

## Supplementary Information

Below is the link to the electronic supplementary material.

Supplementary file1 (DOCX 17 kb)

Supplementary file2 (DOCX 25 kb)

Supplementary file3 (DOCX 39 kb)

Supplementary file4 (DOCX 15 kb)

## Data Availability

The data will be made available on request from the corresponding author.
