# Correction: Neural autoantibodies in psychiatric disorders are associated with antibodies against viral pathogens: a retrospective study of 619 patients

**DOI:** 10.1007/s00702-025-02973-5

**Published:** 2025-07-21

**Authors:** Niels Hansen, Vincent Buschatzky, Anne Katharina Bastin, Kristin Rentzsch, Bianca Teegen, Daniel Luedecke, Thomas Skripuletz, Hannah Benedictine Maier, Stefan Bleich, Jürgen Gallinat, Hermann Esselmann, Ildiko Rita Dunay, Inga Zerr, Dirk Fitzner, Jens Wiltfang, Alexandra Neyazi, Björn Hendrik Schott, Berend Malchow, CAP (Cerebrospinal Fluid Analysis in Psychiatry) consortium

**Affiliations:** 1 Department of Psychiatry and Psychotherapy, University Medical Center Göttingen, Von-Siebold-Str. 5, 37075 Göttingen, Germany; 2 Clinical Immunological Laboratory Prof. Stöcker, Groß Grönau, Germany; 3 Department of Psychiatry and Psychotherapy, University Hospital Hamburg Eppendorf, Hamburg, Germany; 4 Department of Neurology, Hannover Medical School, Hannover, Germany; 5 Department of Psychiatry, Social Psychiatry and Psychotherapy, Hannover Medical School, Hannover, Germany; 6 Institute for Inflammation and Neurodegeneration, Otto-Von-Guericke-University Magdeburg, Magdeburg, Germany; 7 Center for Behavioral Brain Sciences, Otto-Von-Guericke-University Magdeburg, Magdeburg, Germany; 8 Department of Neurology, University Medical Center, Göttingen, Germany; 9 German Center for Neurodegenerative Diseases (DZNE), Von-Siebold-Str. 3a, 37075 Göttingen, Germany; 10 Neurosciences and Signaling Group, Institute of Biomedicine (iBiMED), Department of Medical Sciences, University of Aveiro, Aveiro, Portugal; 11 Department of Psychiatry and Psychotherapy, Otto-Von-Guericke-University Magdeburg, Magdeburg, Germany; 12 Leibniz Institute for Neurobiology, Magdeburg, Germany

**Correction: Journal of Neural Transmission**

10.1007/s00702-025-02943-x

In the original version of this article, the author’s name Jens Wiltfang was incorrectly written as Jens Wilftang and the affiliation ‘Department of Psychiatry and Psychotherapy, Otto-Von Guericke-University Magdeburg, Magdeburg, Germany’ for author Björn Hendrik Schott was missing.

