# Supplementary material for: Neural autoantibodies in psychiatric disorders are associated with antibodies against viral pathogens: a retrospective study of 619 patients
Source: J Neural Transm (Vienna). 2025 May 17;132(7):1063–74. doi: 10.1007/s00702-025-02943-x (PMC12208994; doi:10.1007/s00702-025-02943-x)
Supplement: Supplementary file 1 — Supplementary file1 (DOCX 17 kb) [file 702_2025_2943_MOESM1_ESM.docx]

**Table 1 supplement: Demographic and data of clinical comorbidities patient groups**

|  | F00-F79  Abs+ | F00-F79  Abs- | Statistics  F00-F79 vs. F00-F79 | F00-09  Abs+ | F00-09  Abs- | Statistics  F00-F09 vs. F00-F09 | F20-F29  Abs+ | F20-F29  Abs- | Statistics  F020-F29 vs. F20-F29 | F30-39  Abs+ | F30-39  Abs- | Statistics  F30-F39 vs. F30-F39 |
| --- | --- | --- | --- | --- | --- | --- | --- | --- | --- | --- | --- | --- |
| Age | 67,6 ± 17,07,  n =115 | 62,97 ± 17,57,  n = 504 | p<0.005^1^ | 72,11 ±  13,61  n= 81 | 71,04 ±  11,38) n=296 | p=0.151^1^ | 46,91 ± 17.42  n=8 | 44,21 ±  16,38  n=57 | p=0.670^2^ | 63,66 ± 14,57  n=19 | 54,31 ± 18,84  n=119 | p=0.065^1^ |
| Sex | f: 54/115, 46,96% | f: 237/504  47,02% | p=1^3^ | f:  39/81  48,15% | f: 152/296 (51,35%) | p=0.618^3^ | f:  3/8  (37,5%) | f:  29/57 (50,88% | p=0.708^3^ | f:  8/19  (42,11%) | f:  41/119  (34,45% | p=0.607^3^ |
| Recent COVID19 infection | 3/115  (2,61%)  n=115 | 12/504 (2,38%)  n=504 | p=0.747^3^ | 2/81  (2,47%)  n=81 | 4/295  (1,35%)  n=295 | p=0.613^3^ | 0/8  (0%)  n=8 | 3/57  (5,26%)  n=57 | p=1^3^ | 0/19  (0%)  n=19 | 4/119  (3,36%)  n=119 | p=1^3^ |
| Diabetes mellitus type 1 or 2 | 21/115  18,26% | 63/503  (12,52%) | p=0.130^3^ | 16/81  (19,75%) | 46/295  (15,59%) | p=0.398^3^ | 1/8  (12,5%) | 3/57  (5,26%) | p=0.417^3^ | 4/19  (21,05%) | 11/119  (9,24%) | p=0.741^3^ |
| Presence of Tumor | 16/115  (13,91%) | 89/504  (17,66%) | p=0.408^3^ | 15/81  (18,51%) | 58/296  (19,59%) | p=0.875^3^ | 0/8  (0%) | 4/57  (7,02%) | p=1^3^ | 2/19  (10,53%) | 24/119  (20,17%) | p=0.527^3^ |
| Rheumatological disease | 8/115  (6,97%) | 14/503  (2,78%) | p=0.037^3,#^ | 6/81  (7,41%) | 13/293  (4,44%) | p=0.264^3^ | 0/8  (0%) | 1/57  (1,75%) | p=1^3^ | 2/19  (10,53%) | 1/118  (0,85%) | p=0.051^3^ |

**Abbreviations**: f = female. Statistics: ^1^ = usage of Mann-Whitney U-test, ^2^ = usage of two sample t-test, ^3^ = Fisher´s exact test. # not significant due to Bonferroni correction.
