# Supplementary material for: Neural autoantibodies in psychiatric disorders are associated with antibodies against viral pathogens: a retrospective study of 619 patients
Source: J Neural Transm (Vienna). 2025 May 17;132(7):1063–74. doi: 10.1007/s00702-025-02943-x (PMC12208994; doi:10.1007/s00702-025-02943-x)
Supplement: Supplementary file 2 — Supplementary file2 (DOCX 25 kb) [file 702_2025_2943_MOESM2_ESM.docx]

| **NEURAL AUTOANTIBODY** | **F00-F79** | **F00-F09** | **F20-F29** | **F30-F39** |
| --- | --- | --- | --- | --- |
| ***Intracellular antigens*** |  |  |  |  |
| Recoverin Serum | 10 | 7 | 2 | 1 |
| Recoverin CSF | 2 | 2 | 0 | 0 |
| Titin Serum | 6 | 4 | 1 | 1 |
| Titin CSF | 2 | 2 | 0 | 0 |
| AP3B2 Serum | 1 | 0 | 1 | 0 |
| AP3B2 CSF | 0 | 0 | 0 | 0 |
| ARHGAP26 Serum | 1 | 1 | 0 | 0 |
| ARHGAP26 CSF | 0 | 0 | 0 | 0 |
| Homer 3 Serum | 1 | 1 | 0 | 0 |
| Homer 3 CSF | 1 | 1 | 0 | 0 |
| CARPVIII Serum | 1 | 1 | 0 | 0 |
| CARPVIII CSF | 0 | 0 | 0 | 0 |
| KCNA2 Serum | 2 | 2 | 0 | 0 |
| KCNA2 CSF | 0 | 0 | 0 | 0 |
| GFAP Serum | 2 | 1 | 0 | 0 |
| GFAP CSF | 0 | 0 | 0 | 0 |
| Myelin Serum | 4 | 3 | 0 | 1 |
| Myelin CSF | 0 | 0 | 0 | 0 |
| ***Cell surface autoantibody*** |  |  |  |  |
| AT1A3 Serum | 1 | 0 | 0 | 0 |
| AT1A3 CSF | 1 | 0 | 0 | 0 |
| IgLON5 Serum | 1 | 1 | 0 | 0 |
| IgLON5 CSF | 0 | 0 | 0 | 0 |
| NF186 Serum | 5 | 4 | 0 | 1 |
| NF186 CSF | 0 | 0 | 0 | 0 |
| NF155 Serum | 1 | 1 | 0 | 0 |
| NF155 CSF | 0 | 0 | 0 | 0 |
| MOG Serum | 3 | 3 | 0 | 0 |
| MOG CSF | 0 | 0 | 0 | 0 |
| Glycine Serum | 5 | 5 | 0 | 0 |
| Glycine CSF | 0 | 0 | 0 | 0 |

**Abbreviations**: AP3B2 = **adaptor related protein complex**3 subunit beta 2, ARHGAP26 =

Rho GTPase activating protein 26, AT1A3 = **ATPase Na+/K+ Transporting Subunit Alpha** 3,

CARPVIII = carbonic anhydrase-related protein **VIII**, CSF = cerebrospinal fluid,

GFAP = glial fibrillary acid protein, MOG = myelin oligodendrocytic protein,

NF155/186 = neurofascin 155 / neurofascin 186
