# Supplementary material for: Neural autoantibodies in psychiatric disorders are associated with antibodies against viral pathogens: a retrospective study of 619 patients
Source: J Neural Transm (Vienna). 2025 May 17;132(7):1063–74. doi: 10.1007/s00702-025-02943-x (PMC12208994; doi:10.1007/s00702-025-02943-x)
Supplement: Supplementary file 3 — Supplementary file3 (DOCX 39 kb) [file 702_2025_2943_MOESM3_ESM.docx]

**Table 3 supplement: Cerebrospinal fluid and blood analysis in patients**

|  | **F00-F79**  **Abs+** | **F00-F79**  **Abs-** | **Statistics** | **F00-F09**  **Abs+** | **F00-F09**  **Abs-** | **Statistics** | **F20-F29**  **Abs+** | **F20-F29**  **Abs-** | **Statistics** | **F30-F39**  **Abs+** | **F30-F39**  **Abs-** | **Statistics** |
| --- | --- | --- | --- | --- | --- | --- | --- | --- | --- | --- | --- | --- |
| **CSF** |  |  |  |  |  |  |  |  |  |  |  |  |
| Cell count /µl (<5 /µl) | 1 ± 2 (n=101) | 1 ± 2 (n = 463) | p=0.208^1^ | 1 ± 2 (n = 70) | 1 ± 2 (n = 279) | p=0.264^1^ | 1 ± 1 (n = 7) | 1 ± 1 (n = 52) | P=0.671^1^ | 1± 3 (n = 17) | 1 ± 3 (n = 104) | p=0.836^1^ |
| Lympho-cytes % | 78.8 ± 14.01 (n=85) | 80.4± 12.5  (n=404) | p=0.448^1^ | 79.8 ± 38.4 (n=56) | 80.5 ± 31.2 (n=250) | p=0.552^1^ | 72.6 ± 29.3 (n=7) | 80.5 ± 32.5 (n=46) | p=0.179^1^ | 78.1 ± 37.2 (n=15) | 80.6 ± 39.1 (n=82) | p=0.768^1^ |
| Mono-cytes % | 16.6 ± 10.3 (n=85) | 15.7 ±  9.2 (n=404) | p=0.647^1^ | 16.6 ± 11.1 (n=56) | 15.8 ± 10.2 (n=250) | p=0.504^1^ | 22.1 ± 13.4  (n=7) | 16.0 ± 10.1 (n=46) | p=0.197^1^ | 14.8 ± 12.3 (n=15) | 15.5± 10.6 (n=82) | p=0.306^1^ |
| Granulocytes % | 6.3 ± 9.0 (n=60) | 6.5 ± 9.8 (236) | P=0.609^1^ | 5.3 ± 6.1 (n=36) | 6.1 ± 6.9 (n=149) | P=0.850 | 7.4 ± 7.3 (n=5) | 5.8 ± 6.8 (n=27) | p=0.544^1^ | 9.4 ± 10.9 (n=11) | 6.8 ± 6.9 (n=45) | p=0.859^1^ |
| Lactat mmol/l | 1,6 ± 0,3(n=98) | 1,6 ± 1,1  (n=461) | p=0.191^1^ | 1,6 ± 0,6 (n=69) | 1,6 ±  0,5 (n=279) | p=0.526^1^ | 1,5 ± 0,6 (n=7) | 1,4 ± 0,4 (n=52) | p=0.066^2^ | 1,53 ± 0,66 (n=15) | 1,5 ± 0,6 (n=102) | p=0.344^2^ |
| Total protein mg/l | 435,91 ± 227,08 (n=101) | 429,34 ± 328,87 (n=463) | p = 0,187^1^ | 441,27 ± 264,46 (n=70) | 460,6 ± 398,70  (n=279) | p=0.547^1^ | 356,29 ±  141,51  (n=7) | 341,84 ± 126,37 (n=52) | p=0.700^2^ | 428,82 ± 203,25 (n=17) | 407,35 ± 231,60 (n=104) | p=0.476^1^ |
| Albumin mg/l | 310,30 ±  204,85 (n=101) | 305,26 ± 260,26 (n=463) | p=0.080^1^ | 312,85 ±  221,92 (n=70) | 327,63  ± 308,98  (n=279) | p=0.499^1^ | 244,00  ±  103,01 (n=7) | 238,60 ± 99,04 (n=52) | p=0.664^1^ | 303,47 ± 157,17 (n=17) | 294,77 ±  192,62 (n=104) | p=0.551^1^ |
| Albumin quotient  Q (CSF/  serum)*10^3^ | 7,66 ±  5,09 (n=101) | 7,68 ± 9,36 (n=463) | p=0.910^1^ | 7,74 ±  5,59 (n=70) | 8,36 ± 11,45 (n=279) | p=0.554^1^ | 6,16 ± 2,72 (n=7) | 5,86 ± 2,40 (n=52) | p=0.705^2^ | 7,70 ± 4,23 (n=17) | 7,15± 4,50 (n=104) | p=0.526^1^ |
| IgG quotient  Q (CSF/  serum)*10^3^ | 3,81 ±  3,46 (n=101) | 3,80  ± 6,17 (n=463) | p=0.834^1^ | 3,95  ±  3,78 (n=70) | 4,16  ± 7,48 (n=2,79) | p=0.772^1^ | 2,85 ±  1,23 (n=7) | 2,82 ±  1,17 (n=52) | p=0.944^2^ | 3,60 ±  2,14 (n=17) | 3,58 ± 3,22 (n=104) | p=0.748^1^ |
| IgA quotient  Q (CSF/  serum)*10^3^ | 2,19 ± 2,71 (n=101) | 2,15 ±  4,26 (n=461) | p=0.844^1^ | 2,25 ±  2,91 (n=70) | 2,31 ±  5,01 (n=278) | p=0.725^1^ | 1,58 ±  0,79 (n=7) | 1,53 ±  0,76 (n=51) | p=0.847^2^ | 2,08 ±  1,44 (n=17) | 2,15 ± 2,91 (n=104) | p=0.944^1^ |
| IgM quotient  Q (CSF/  serum)*10^3^ | 0,97 ±  3,00 (n=60) | 0,96 ±  3,84 (n=298) | p=0.305^1^ | 1,16 ±  2,62 (n=43) | 1,10 ±  3,51 (n=172) | p=0.347^1^ | 0,36 ± 0,19 (n=3) | 0,41 ±  0,33 (n=37) | p=0.504^1^ | 0,46 ±  0,33 (n=12) | 1,03 ± 2,65 (n=72) | p=0.929^1^ |
| Intrathecal IgG synthesis | 11/103 (10.7%) | 33/462 (7.1%) | p=0.224^3^ | 10/71 (14.08%) | 5/280 (1.79%) | p<0.001^3^ | 0/8 (0%) | 0/52 (0%) | p=1^3^ | 0/17  (0%) | 8/103 (7.8%) | p=0.599^3^ |
| VZV quotient | 4.82 ±  6.85 (n=84) | 3,72 ±  7,10 (n=366) | p=0.178^1^ | 5,39 ±  7,10 (n=59) | 4,11 ±  7,90 (n=220) | p=0.104^1^ | 2,77 ± 1,57 (n=3) | 2,61 ±  1,5 (n=41) | - | 3,36 ±  2,41 (n=13) | 3,48 ±  2,86 (n=84) | p=0.626^1^ |
| Measles quotient | 3,62 ±  3,62 (n=82) | 3,40  ± 5,97 (n=355) | p=0.560^1^ | 3,63  ±3,63 (n=59) | 3,55 ±  6,36 (n=233) | p=0.802^1^ | 2,42 ±  ; 1,45 (n=5) | 2,58 ±  1,65 (n=34) | p=1^1^ | 4,00  ± 2,84 (n=12) | 3,47 ±  3,47 (n=80) | P=0.394^1^ |
| Rubella quotient | 3,87 ± 4,54 (n=86) | 3,43 ± 5,45 (n=355) | p=0.921^1^ | 3,98  ± 4,71 (n=60) | 3,68  ±5,86 (n=211) | p=0.499^1^ | 2,65 ±  1,20 (n=7) | 2,67 ±  1,64 (n=64) | p=0.675^1^ | 3,79±  2,71 (n=14) | 3,40± 3,24 (n=81) | p=0.475^1^ |
| HSV quotient | 3,97 ±  3,6 (n=79) | 3,70 ±  4,62 (n=327) | p=0.533^1^ | 4,19 ±  3,84 (n=60) | 3,89 ±  4,96 (n=205) | p = 0,483^1^ | 3,04 ± 1,69 (n=3) | 3,03 ±  1,90 (n=38) | - | 3,54 ±  2,74 (n=12) | 3,69 ±  2,61 (n=66) | p=0.502^1^ |
| EBV quotient | 2,97 ±1,68 (n=21) | 4,2 ±  8,79 (n=105) | p=0.565^1^ | 2,98 ±  1,01 (n=9) | 5,82 ±  5,23 (n=35) | p=0.727^1^ | 2,67 ±  1,51 (n=3) | 2,51 ±  1,35 (n=20) | - | 3,82 ±  2,25 (n=6) | 3,83 ±  3,06  (n= 42) | p=0.629^1^ |
| t-tau  pg/ml  (<450 pg/ml ) | 303,43 ±  174,71 (n=90) | 330,0 ±  255,29 (n=393) | p=0.825^1^ | 332,2 ±  210,72 (n=67) | 389,37  ±  285,98 (n=269) | p=0.287^1^ | 173,33  ±  99,87(n=3) | 184,38  ± 108,67 (n=24) | - | 224,87  ±  138,95 (n=15) | 184,74 ±  128,22 (n=81) | p=0.063^1^ |
| P-tau181 pg/ml  (<61 pg/ml) | 73,68  ±  43,51 (n=91) | 77,23 ±  55,29 (n=406) | p=0.945^1^ | 81,46 ±  = 53,07 (n=67) | 88,67  ±  63,63 (n=276) | p=0.386^1^ | 48,33 ±  26,54 (n=3) | 50,96 ±30,36 (n=24) | - | 49,87  ±24,69 (n=16) | 49,15 ±  29,44 (n=86) | p=0.487^1^ |
| Aβ1-42  pg/ml  (>450 pg/ml) | 947,62  ±407,0 (n=92) | 1020,67  ± 469,02 (n=406) | p=0.062^1^ | 868,88  ±  451,84 (n=67) | 917,44  ±  491,34 (n=276) | p=0.739^1^ | 1338,75  ±  791,22 (n=4) | 1280,00  ±  753,74 (n=27) | - | 1177,56  ±  597,71 (n=16) | 1204,87  ± 646,48 (n=83) | p = 0.805^2^ |
| Ratio Aβ1-42/1-40x 10  (>0.5) | 0.89 ± 0.6 (n=92) | 0.97 ± 0.6 (n=405) | p=0.044^1^ | 0.83± 0.61 (n=67) | 0.9 ± 0.5 (n=275) | p=0.706^1^ | 1.04 ± 0.59 (n=4) | 1.35 ± 0.73 (n=27) | - | 1.12 ± 0.55 (n=16) | 1.22 ± 0.73 (n=83) | p=0.624^1^ |
| Aβ1-40 | 11906,66 ± 4298,45 (n=92) | 11735,07  ± 4676,91 (n=407) | p=0.584^1^ | 11997,64  ± 6111,21 (n=67) | 12019,21  ± 5466,13 (n=275) | p=0.878^1^ | 12552,75  ± 6904,45(n=4) | 11348,78  ± 6992,87 (n=27) | - | 11302,81  ± 5659,54 (n=16) | 10766,18 ±  5681,52(n=85) | p=0.536^1^ |
| S100 µg/l  (<2.7 µg/l) | 2,96 ±  1,24 (n=49) | 2,82 ±  1,16 (n=167) | p=0.720^1^ | 2,68 ±  1,51 (n=36) | 3,16 ±  1,65 (n=99) | p=0.066^1^ | 2,74 ±  1,51 (n=3) | 2,12 ±  1,11 (n=19) | - | 4,06 ±  2,255 (n=7) | 2,39 ±  124 (n=42) | p=0.007^1^ |
| NSE ng/ml  (<30 ng/ml) | 22,06 ±  5,40 (n=49) | 21,11 ±  7,21 (n=170) | p=0.311^1^ | 21,67 ±  11,37 (n=36) | 23,10  ±  11,81 (n=101) | p=0.297^2^ | 21,89 ±  12,00 (n=3) | 16,18 ±  8,17 (n=19) | - | 23,08 ±  11,92 (n=7) | 18,73 ±  9,73 (n=43) | p=0.083^2^ |
| Blood CSF barrier disturbance % | 31/115 (26.9%) | 107/462 (23.2%) | p=0.394^3^ | 20/71  (28.2%) | 23/281 (8.2%) | p<0.05# | 2/8 (25%) | 13/52 (25%) | p=1^3^ | 6/17 (35.3%) | 28/103 (28%) | p=0.563^3^ |
| **Blood** |  |  |  |  |  |  |  |  |  |  |  |  |
| NSE  ng/ml  (<30 ng/ml) | 20,13 ±  6,72 (n=57) | 18,78 ±  5,70 (n=178) | p=0.229^1^ | 19,19 ±  10,59 (n=43) | 18,62 ±  9,35 (n=102) | p=0.718^1^ | 25,42 ±  12,15 (n=2) | 19,22 ±  9,21 (n=20) | - | 22,33 ±  12,60 (n=9) | 18,90 ±  10,20 (n=47) | p=0.153^1^ |
| S100  µg/l  (<0.15 µg/l) | 0,16 ±  0,56 (n=55) | 0,11 ±  0,23 (n=174) | p=0.365^1^ | 0,18 ±  0,47 (n=41) | 0,09 ±  0,05 (n=99) | p=0.304^1^ | 0,06 ±  0,03 (n=2) | 0,09 ±  0,05 (n=20) | - | 0,09 ±  0,06 (n=9) | 0,16 ±  0,29 (n=46) | p=0.507^1^ |

**Abbreviations**: Aβ1-40 = amyloid beta 1-40, Aβ1-42 = amyloid beta 1-42, CMV = choriomeningitis virus, CSF = cerebrospinal fluid, EBV = Ebstein bar virus, HSV = herpes simplex virus, IgA = immunoglobulin A, IgG = immunoglobulin G, IgM = immunoglobulin M, NSE = neuron specific enolase, Ptau181 = phosphorylated tau protein 181, Ratio Aß1-42/-1-40 = amyloid beta 1-42/ amyloid beta 1-40, t-tau = total tau protein, VZV = Varizella zoster virus. The non-pathological reference values from the Neurochemistry laboratory from the Department of Neurology, University Medical Center Göttingen are indicated in brackets. Statistics: ^1^ = usage of Mann-Whitney U-test, ^2^ = usage of two sample t-test, ^3^ = Fisher´s exact test. # not significant due to Bonferroni correction.
