# Supplementary material for: Neural autoantibodies in psychiatric disorders are associated with antibodies against viral pathogens: a retrospective study of 619 patients
Source: J Neural Transm (Vienna). 2025 May 17;132(7):1063–74. doi: 10.1007/s00702-025-02943-x (PMC12208994; doi:10.1007/s00702-025-02943-x)
Supplement: Supplementary file 4 — Supplementary file4 (DOCX 15 kb) [file 702_2025_2943_MOESM4_ESM.docx]

**Table 4 supplement: Patient numbers of anti-viral specific antibody indices**

| ASI type and NAB positivity | F00-F79,  n | F00-F09,  n | F20-F29,  n | F30-F39,  n |
| --- | --- | --- | --- | --- |
| VZV NAB+ | 84 | 59 | 5 | 13 |
| VZV NAB- | 366 | 220 | 41 | 84 |
| HSV NAB+ | 79 | 60 | 3 | 12 |
| HSV NAB- | 327 | 205 | 38 | 66 |
| Rubella NAB+ | 86 | 60 | 7 | 14 |
| Rubella NAB- | 355 | 211 | 43 | 81 |
| Measles NAB+ | 82 | 59 | 5 | 12 |
| Measles NAB- | 355 | 222 | 34 | 80 |
| EBV NAB+ | 21 | 9 | 3 | 6 |
| EBV NAB- | 105 | 35 | 20 | 42 |

**Abbreviations**: ASI = anti-viral specific antibody index, EBV = Ebstein bar virus, HSV = herpes simplex virus, IgG = immunoglobulin type G, NAB = neural autoantibody, NAB+ = neural autoantibody positivity, NAB- = neural autoantibody negativity, n =number of patients, VZV = Varizella zoster virus. The ASI refers to IgG.
